# Arts and sciences

**DOI:** 10.3389/fnhum.2013.00008

**Published:** 2013-01-31

**Authors:** Ofer Lellouche

**Affiliations:** Painter and SculptorTel Aviv, Israel

The idea of bringing together the arts and sciences is not new. In almost every age, these two disciplines have had a difficult and fragile relationship. Where does this mutual fascination come from? What are the limits of this dialog? How can we avoid misunderstanding, and manage this encounter in a productive way?

As an artist, I am often asked “Why do you need this?” Actually my answer is that I do not know. I do not need it to paint, nor to ameliorate my art, and even not to reach new directions of exploring. Five years ago, I participated in a seminar regrouping 10 leading brain researchers with 10 leading artists. For three days, artists and scientists spoke about their work. It seemed to me like two parallel monologs that can never meet; scientists spoke about science and artists spoke about their art.

As a painter, I was fascinated to discover during the seminar that what we artists always knew, in an intuitive way, had a theoretical basis. I could better explain, for instance, why there is a huge difference between painting from nature and painting from photography.

But, naturally, this symposium did not influence my way of painting; for me, as for many other artists, the moment we create must be completely intuitive. It slightly resembles the moment the goalkeeper of a football team reacts in front of a penalty shot: a mixture of extreme concentration and of emptiness of the mind. But in order to arrive to this second where everything is possible, he has to suffer many trainings. In a way, the seminar was for me part of this training.

The language of art is a highly sophisticated one, and it is obvious why it is a privileged subject for sciences of the brain. Nevertheless, sciences, or any other rigorous communicative language, cannot explain art. The first sentence of Zeki (1999) in his book *Inner Vision* is, “This is not so much a book about art, it is more a book about brain.” This impossibility is not due to a provisory lack of information, but because of the very different structure of the language of art and science.

What do we expect in the contemplation of a piece of art? For us, moderns, it certainly does not have a magical function (like Egyptian Art, for instance, a function which has a real influence on the real world). When art lovers are asked to better define why they need art, they speak of pleasure. But what kind of pleasure? Is it the same kind of pleasure that one derives from a good meal? Should painting be studied by scientists as gastronomy is?

This comparison hurts the artists (Although, alas, art critics and restaurant critics share the same page of the newspapers!). When people are asked to be more precise about the word “pleasure,” they speak of “emotion,” they evoke the feeling of “diving” into the piece, to “forget themselves,” to identify themselves with the subject, they speak of a “dream awakened,”—all are feelings that you could not have while sitting in front of a plate of seafood. A piece of art makes us dream.

Of course, a piece of art also delivers an esthetic pleasure in the “gastronomic” sense of the word, but this is secondary. In what way does a piece of art make us dream? Why can we not stop looking at the Madonna by Raphael, while the (almost) same painting by a different artist leaves us indifferent? Why does the Montagne Sainte-Victoire painted by Cézanne makes us dream, while a photograph of it just looks like a mountain? Art is not a representation of nature in a beautiful way; It is a different kind of signification. There is a deep difference between the language of art and the language of everyday.

*The language-of-everyday* is based upon the assumption that you can understand what is said. The more accurate it is, the more communicative it becomes (For instance, in writing these lines, I make great effort to be *as clear as possible*). Sometimes, perfectly understanding this language is a question of life and death; if you do not understand the signification of a red light, for example, you might have a car accident. The language of law and the language of the sciences should be as precise as possible. The most minimalist expression of this language is the pictogram. When you see a sign representing very schematically a man and a woman—often just a circle representing the head, a triangle for a skirt and two rectangles for the trousers—you understand that this signifies the restrooms.

But just suppose this sign is found by future archeologists who have no understanding of its *meaning*; they will understand it through associations, they might hang this piece in a museum of art next to Adam and Eve by Durer, a Fang wooden sculpture of a couple, an Egyptian painting of Nout (the deity of sky) and Geb (the deity of earth), Joseph and Miriam by Rembrandt, and many other pieces of “art.”

There is, I think, a great difference between the language of art and the pictogram (or the-language-of-everyday). The language of the pictogram functions through the understanding of the negation, the language of art functions through associations and ignores negation.

One pictogram is enough to describe the universe. Take, for example, the pictogram of a tree. You can split the universe into “tree” and “not a tree.” It would be a very poor language but it would be, thanks to the negation, enough to describe the universe. But the painting of a tree means something else.

Many years ago, I painted my 3-year-old son and my 5-year-old daughter beneath a huge palm tree. When my son explained the painting, he said, “This is my sister. This is me. And this tree is my father.” In a sense, he understood that the question is not “Is this a tree or not a tree?” but about the different associations the tree can evoke.

I would like to be more precise about this notion of association and give a few examples:

1. Association to another piece of art: those three trees etched by Rembrandt remind us of the three crosses of the crucifixion, they remind us also of the hair of this self-portrait. Something of the “sense” of the crucifixion affects the “sense” of the landscape. Something of the curled hair colors the “signification” of the trees.
2. Associations within a painting: when Cézanne says that the shoulders of the women must be connected with the curb of the mountains, something of the “sense” of the mountain affects the “sense” of the women and vice versa. Those influences of sense occur all the time in a piece of art, and are absent in a “banal” photograph which has been “decorated” with artistic effects.
3. The association can work in the direction of the world to the painting, like in the case of my 3-year-old son, but it can also work in the other direction: from the painting to the world. I always say that for me the most important place in a museum is the windows: a visit to the Louvre, and through a window I can see trees, cars, buses … I walk a few steps and stand in front of a Poussin … a few more steps, and I reach another window, but the trees “have changed,” they look like the trees in the painting. To me, probably the most important “function” of art is “to change our vision of nature.” An exhibition has a signification when I go outside and the street has changed.
  Art accompanies us for a long time after we have exited the museum. A few months ago, while immobilized for a treatment of acupuncture, I did the following experiment: i tried to listen to the noises surrounding me as if they were a piece of music: the cars, the steps, the voices, the birds, the wind in the trees … (The day before, I listened to a contemporary music concert that used the same noises). I was listening as if it “had a sense,” as if it was a piece of music written by some composer. After only a few minutes, I was so exhausted that I could not go on. Being a painter, this is what I am doing almost all of the time with my sense of vision, and in a natural way, without difficulty. My many years of practice allow me to “see” the world as if it were a painting.
4. The list of the associations art can provide is as long as all fields of human activities: music, poetry, philosophy, politics ….

**Figure 1:**
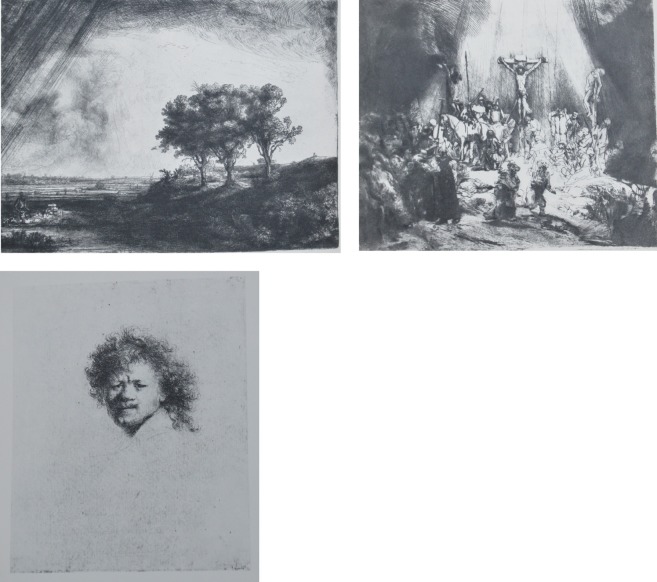
**Rembrandt VanRijn, (up left) Three trees, etching with dry point and engraving. (up right)** The three cross, etching with dry point and engraving, **(down left)** Self portrait, etching with dry point and engraving.

Language of art functions in a completely different way than *the-language-of-everyday*. Moreover, the language of everyday is much too “poor” to describe the language of art. Kantor demonstrates why a group (a,b,c, …) is always “*smaller*” than a group of the associations of those elements (a, ab, abc, ac, …). It might be possible to use this demonstration to show why our ordinary language is too poor to describe a piece of art (which functions through associations). To me, it seems intuitively true.

Poets and lawyers speak two completely different languages with different rules even though they use the same raw materials of vocabulary. This is why it is a non-sense to try to translate a piece of poetry into its own language, and why it is necessary to translate it infinitely into others, while a legal document needs only be translated once. Furthermore, attempts to explain art using the language of everyday freezes the streams of infinite associations.

The notion of “sense” is completely different when it refers to a piece of art. The question “what does it mean?”—which includes in ordinary language the possibility of saying “what it does not mean”—has no meaning when it deals with art. One of my paintings was described by two prominent and sensitive critics as “a hymn to life” and as “a march toward death.” I did not feel any contradiction between those two statements. Hamlet could be a young blond virgin actor, or a fat, unshaven, bisexual 45-year-old-man. This text makes us dream. This is why there is a need to come back to it again and again… Art is a paradox, in the sense that you are not able to say if it is right or wrong.

When I am asked that question, “*What does it mean*?” I always answer “everything.” A real, a deep, a great piece of art contains, through an infinite number of associations, the whole world.

One of the most common misunderstandings about art is to think that it is composed of “sense” (a pictogram) decorated with “beauty.” In this way of seeing, the painting of Cézanne would be the painting of a mountain, but represented in a “beautiful” way, like a beautifully “*designed*” mountain. There are computers programs that allow us to transform a banal photograph into something that looks like a painting. Too often, the painter is considered almost as if he or she is a very sophisticated program of this kind, and this is wrong. To me, a piece of art is not “sense” + “beauty,” or just “beauty.” It is another kind of sense.

Art makes us dream, a pictogram does not. A painting of a tree is not a pictogram of a tree with the addition of “beauty.” Art, as well as dreams, functions by associations and ignores negation. Like in a dream, the objects or the forms, because of the very special structure of the work of art, undergo transformations, metamorphoses that do not hurt our logic.

This is also the reason why there is progress in science and not in art. The physics of Newton becomes a particular detail in the theory of Einstein. On the contrary, Cezanne, by creating new associations with the work of Poussin, makes it richer (Moreover, the work of Poussin enriches the work of Cezanne. Time functions in both directions). A new piece of art does not render an earlier piece of art irrelevant or old-fashioned; rather, it enriches it as it does with all other existing pieces. While listening to a quartet of Bartok, you can hear how it dialogs with Beethoven, with Bach, or with Hungarian folk music. In a way, we could say that linear history has, in this context, no meaning. Therefore, whereas the ideal of science is to develop a language which should be more and more concise, the language of art is becoming wider and wider. The language of sciences is convergent; the one of art is divergent.

This makes any description of art very limited. If you were one day in the presence of two painters speaking of art, you would probably be surprised at how clumsy the dialog can be. Using pantomime, or very vague expressions like “it works” or “it does not work,” using concepts from other disciplines like “it is too sweet!” or “too heavy,” or taken from music like “rhythm,” “tonality,” “accord,” “major or minor” (It works also the other way: i have assisted at a violin master-class with Shlomo Mintz where he used concepts taken from painting, like “line,” “colorful,” “black and white,” “blurry”…!).

It is understandable, why art, which requires a highly sophisticated activity of the brain, interests scientists. Not as an effort to understand art better, but for the needs of scientific research. It is less understandable why artists need sciences.

It could be that artists are just interested in all fields of human behavior. They “use” scientific theory as a source of inspiration and then throw it away. At some time they are interested by the theory of the decomposition of light, and they create Impressionism, at other times by the theory of relativity, and they create Futurist paintings, etc. This, without any linear coherence to the theories they refer to. It is just a toy they can play with, and then leave.

It could also be that science and art are both linked to some “deep philosophy” of the time. It is fascinating to see, for instance, that the publication of the poem “Un coup de dés jamais n'abolira le hasard” by Stéphane Mallarmé, was published at the same time as Poincarré studies on the hazard, while there were no known connections between the two men. For some obscure reason, some idea circulates at a certain time and challenges both scientists and artists.

As I wrote above, the seminar on Art and Brain was—and still is—a part of my “training” as an artist. It allowed me to understand better the way in which vision functions, but the main lesson of this encounter was the idea of mapping. The idea of dividing the brain activity into zones which have different functions and work as a net of inter-references, has some similarity with the activities of the young generation of artists, mixing in the same exhibition video art, heterogeneous drawings (figurative, abstracts, pictograms), installations, performances, etc. It seems to me that this way of activity, also parallels the way we use the internet, and in some strange and remote way, the new conception of the world as a net of multi-cultural, multi-national, multi-religious identities. This idea of a dynamic “map” is, today, in the *spirit of the time*, and it seems that the Hegelian conception of history of art is transformed into a more dynamic conception of the “geography” of art. Instead of historical exhibitions, there are more and more museums showing side by side African sculptures, Egyptian art, oil paintings of the seventeenth century, with contemporary art. This might be the *spirit of the time* both for science and for art.
